# Giant strain with ultra-low hysteresis and high temperature stability in grain oriented *lead-free* K_0.5_Bi_0.5_TiO_3_-BaTiO_3_-Na_0.5_Bi_0.5_TiO_3_ piezoelectric materials

**DOI:** 10.1038/srep08595

**Published:** 2015-02-26

**Authors:** Deepam Maurya, Yuan Zhou, Yaojin Wang, Yongke Yan, Jiefang Li, Dwight Viehland, Shashank Priya

**Affiliations:** 1 Bio-inspired Materials and Devices Laboratory (BMDL), Center for Energy Harvesting Materials and Systems (CEHMS), Virginia Tech, 24061 USA; 2 Department of Materials Science and Engineering, Virginia Tech, Blacksburg, VA 24061, USA

## Abstract

We synthesized grain-oriented lead-free piezoelectric materials in (K_0.5_Bi_0.5_TiO_3_-BaTiO_3_-xNa_0.5_Bi_0.5_TiO_3_ (KBT-BT-NBT) system with high degree of texturing along the [001]c (c-cubic) crystallographic orientation. We demonstrate giant field induced strain (~0.48%) with an ultra-low hysteresis along with enhanced piezoelectric response (d_33_ ~ 190pC/N) and high temperature stability (~160°C). Transmission electron microscopy (TEM) and piezoresponse force microscopy (PFM) results demonstrate smaller size highly ordered domain structure in grain-oriented specimen relative to the conventional polycrystalline ceramics. The grain oriented specimens exhibited a high degree of non-180° domain switching, in comparison to the randomly axed ones. These results indicate the effective solution to the lead-free piezoelectric materials.

Piezoelectric materials find myriad applications in sensors, actuators, and energy harvesting devices. However, the temperature range of operation of these devices is limited by the Curie temperature (*T*_c_), or in some cases depoling temperature (*T*_d_), as the piezoelectric systems lose their functional response above these transition temperatures. Currently, most of the devices use Pb(ZrTi)O_3_ (PZT) based materials due to better piezoelectric responses and high Curie temperatures (~390°C)^1^ Moreover, the piezoelectric response of PZT based materials can be easily tuned by substituents (e.g. soft and hard types) with minor shift in *T*_c_ towards lower temperatures. However, in the case of lead-free piezoelectrics, substituents have not been widely reported to be as effective, and often they have been found to be associated with shifting of *T*_c_ and *T*_d_ towards lower temperatures (Fig. 1a)^2^,^3^,^4^,^5^. Specifically, the low value of *T*_c_ and *T*_d_ are detrimental for the application of these piezoelectric materials as actuators. Many efforts have been made to demonstrate enhanced piezoelectric responses in these systems, however due to lower *T*_c_/*T*_d_ values (Table 1)^6^,^7^,^8^,^9^,^10^,^11^, practical applications have been limited. Often, high values of strain have been found to be accompanied by large hysteresis (Fig. 1b)^6^,^7^,^8^,^9^,^10^,^11^, which further restricts applications as piezoelectric actuators. Presently, in the functional ceramics community, there is no problem more important than finding a replacement for lead-based piezoelectrics. In the present work, we have successfully demonstrated giant *E*-field induced strain with a ultra-low degree of hysteresis, along with enhanced low field piezoelectric response (*d*_33_) and high temperature stability (>160°C) in lead-free piezoelectric system exhibiting their high technological relevance.

Recently, K_0.5_Na_0.5_NbO_3_ (KNN), Bi_0.5_Na_0.5_TiO_3_ (NBT), and Ba(Ti_0.8_Zr_0.2_)O_3_-(Ba_0.7_Ca_0.3_)TiO_3_ based lead-free piezoelectric ceramics have attracted much attention^12^,^13^. KNN based materials utilize Nb_2_O_5_ for synthesis, which is expensive and would notably increase production costs. Also, KNN based materials are sensitive to moisture and have sintering issues due to the volatile nature of alkali elements^12^. Ba(Ti_0.8_Zr_0.2_)O_3_-(Ba_0.7_Ca_0.3_)TiO_3_ based materials exhibit a high piezoelectric response, however a low Curie temperature (<93°C) restricts their use in practical application^13^. Alternatively, Na_0.5_Bi_0.5_TiO_3_ (NBT) based piezoelectric materials have been considered as potential candidates to replace PZT based piezoelectric materials^14^. Various researchers have attempted to improve the piezoelectric response of NBT-based lead-free piezoelectrics by compositional modification, however, no appreciable improvement in properties has been observed. Moreover, most of the time, enhanced piezoelectric properties were found to be accompanied with a lower depoling temperature (*T*_d_)^2^,^3^,^4^,^5^, limiting their temperature range of operation (Fig. 1) as discussed earlier. In our previous work, we have reported large improvements in the piezoelectric response in textured NBT-BT near the morphotropic phase boundary (MPB)^15^. However, the *T*_d_ of the NBT-BT grain oriented ceramics was ~90°C^15^, which restricts their usage in practical applications as sensors and actuators due to the limited temperature range of operation defined by lower value of *T*_d_. As discussed earlier, the lower value of *T*_d_ has been found to result in an additional complexity in terms of increased non-linearity in *E*-field induced strain characteristics (Table 1 and Fig. 1b), which imposes additional constraints. In order to address this major challenge of low T_d_, we provide a new pathway by invoking ternary phase boundaries. K_0.5_Bi_0.5_TiO_3_ (KBT) with tetragonal structure has been shown to provide a MPB with rhombohedral Na_0.5_Bi_0.5_TiO_3_ (NBT) and tetragonal BaTiO_3_ (BT) in a ternary phase system^16^. The addition of KBT to the binary NBT-BT system was found to exhibit a long range tetragonal structure while moving away from the MPB^2^. Selecting a non-MPB composition in the ternary KBT-BT-NBT system especially on the tetragonal side results in higher *T*_d_ and *d*_33_ as compared to binary NBT-BT composition^16^ This result can be explained by considering the effect of substitution of larger potassium (K) ion on the A-site of NBT-BT. The K^+^ (1.64 Å in 12 fold coordination) ion results in increased repulsive interaction with B-site (Ti^4+^) ion that influences the correlated off-centering and assists the long range tetragonal polar ordering^14^,^17^. In our previous study, we have shown that the substitution of larger cation on A-site can increase the transition temperature and polar ordering due to strain effect^17^. We think that the higher *T*_d_ in the ternary phase system can be attributed to the strain effect due to the presence of larger *K*^+^ ion on A-site^17^. If the composition is slightly away from the MPB towards tetragonal side, the strain through non-180° domain switching is only expected in polycrystalline ceramics having <001>_c_ texture^18^. Our approach based upon the grain orientation allows the composition on the tetragonal side of the ternary phase diagram of KBT-BT-NBT system to maintain higher *T*_d_ with enhanced piezoelectric response by taking advantage of electrocrystalline anisotropy. This study describes the fundamental aspects of the grain-oriented K_0.5_Bi_0.5_TiO_3_-BaTiO_3_-Na_0.5_Bi_0.5_TiO_3_ (KBT-BT-NBT) ceramics with a high temperature stability (>160°C), high longitudinal piezoelectric coefficient (*d*_33_ = 190 pC/N), and a giant strain (0.48%) with an ultra-low degree of hysteresis. We have utilized transmission electron microscopy and piezoresponse force microscopy measurements to explain the basis for this excellent performance by understanding the modulation of domain structures.

## Results and Discussion

### Grain-oriented KBT-BT-NBT polycrystalline ceramics

For texturing, we selected a (1-x)(K_0.5_Bi_0.5_TiO_3_-BaTiO_3_)-xNa_0.5_Bi_0.5_TiO_3_ composition with KBT: BT = 2:1 and x = 0.8 (denoted as KBT-BT-NBT) that was away from the MPB and that had a higher depoling temperature (~200°C). In order to obtain a high degree of texturing with high piezoelectric response, we optimized the processing conditions including time and temperature. To investigate phase formation and degree of texturing, we recorded room temperature XRD-scans for various specimens with different degrees of texture. A splitting in the {200}_c_ Bragg refelection confirmed the crystallization in to a tetragonal phase (Fig. 2a) with polarization vector lying along the <001>_c_ direction. The intensity of the (110)_c_ peak was the highest for radomly axed ceramics. With increasing degree of texture, the intensity of (110)_c_ bragg reflections was found to decrease alongwith the increase in the intensity of (001)_c_ and (002)_c_ reflections. This demonstrates that the specimen had a preferred crystallographic orientation along <001>_c_. In order to better charecterize the degree of texture, we obtained pole figures about (002)_c_ (Fig. 2b). Fig. 2b shows a radially symmetric grain distribution, indicating a fiber texture^19^. The pole figure data confirmed the presence of high degree of texturing along <001>_c_ (Fig. 2b). Figs. 2c-d show cross-section SEM micrographs of the etched surface of non-textured and textured (93%) specimens, respectively. The grain size of non-textured specimens was ~1 μm, however, samples having 93% degree of texture had grain size ~5 μm. Please note the presence of a seed in the middle of the textured grains. Next, we obtained TEM images to reveal the interfaces in textured specimen. Fig. 2e shows a high resolution TEM (HR-TEM) image of textured grain and the seed interface. The inset in this figure at low magnification illustrates an interfacial region. Please note the defect free interface, which may be attributed to a small lattice mismatch between KBT-BT-NBT textured grains and the BT seed templates. The defect-free coherent interface is an important factor for achieving enhanced piezoelectric response^15^. In NBT based systems, a liquid phase mechanism dominates the sintering process, facilitating mass transport during growth of textured grains^15^,^20^. The growth of textured grains could be explained by an Ostwald ripening-like process, where larger grains (seed crystals) grow at the expense of smaller ones (matrix grains) because of a difference in surface energy^21^. During growth, dissolution of the polycrystalline matrix occurs, which subsequently precipitates on the lowest energy surface (001)_c_ of the template^15^. This process of growth of grain oriented ceramics, incorporating a dissolution and precipitation mechanism, represents a hetroepitaxial growth process where KBT-BT-NBT textured grains grow on the BT seed templates. The practical growth rate of grain oriented ceramics was low. Therefore, to achieve higher degrees of texture, we used prolong sintering times up to 50 h, which resulted into larger grain sizes in the textured specimens.

### Enhanced piezoelectric response with high temperature stability

Fig. 3a shows *d*_33_ as a function of the degree of texturing along the (001)_c_ axis for KBT-BT-NBT. From this plot it can be seen that the value of *d*_33_ increases rapidly with increase in the degree of texturing. Specimen having a 93% texturing had *d*_33_ ~ 190 pC/N. This is quite interesting, as the NBT based systems generally exhibit enhanced piezoelctric responses with lower depoling temperatures, which limits the temperature range of operation of the devices based on these piezoelectric materials. In order to investigate the temperature dependent phase transition behaviour of NBT systems, we measured the dielectric response as a function of temperature for textured and non-textured specimens. Figs. 3b-c show the temperature dependence of the relative permittivity and loss tangent at various frequencies for non-textured and textured samples. The textured specimen had a depoling temperature *T*_d_ ~ 165°C as marked in Fig. 3c. The relative permittivity of the textured samples appeared to be lower than that of the non-textured one due to the lower dielectric constant of the BT seed (present inside the textured ceramic body) along the [001]_c_^22^. However, the values of the loss tangents were similar for both samples below their depoling temperatures, which is important for practical applications. At high temperature (>300°C) and lower frequency (1 kHz), the higher rate of increase in the relative permittivity and loss tangent, in textured sample, can be attributed to the presence of space charge polarization at the interface of textured grain and seed template. Fig. 3d shows *d*_33_ as a function of temperature. It can be seen that *d*_33_ value of the textured sample remained almost unchanged upto 165°C, reflecting the high temperature stability. However, for non-textured sample, *d*_33_ remained stable upto >200°C before dropping to a lower value. The temperature at which *d*_33_ drops suddenly is related to the depoling temperature of the corresponding specimen.

### Unipolar strain versus *E*-field (*S-E*) and Polarization versus *E*-field (*P-E*) plots

**Figure 1:**
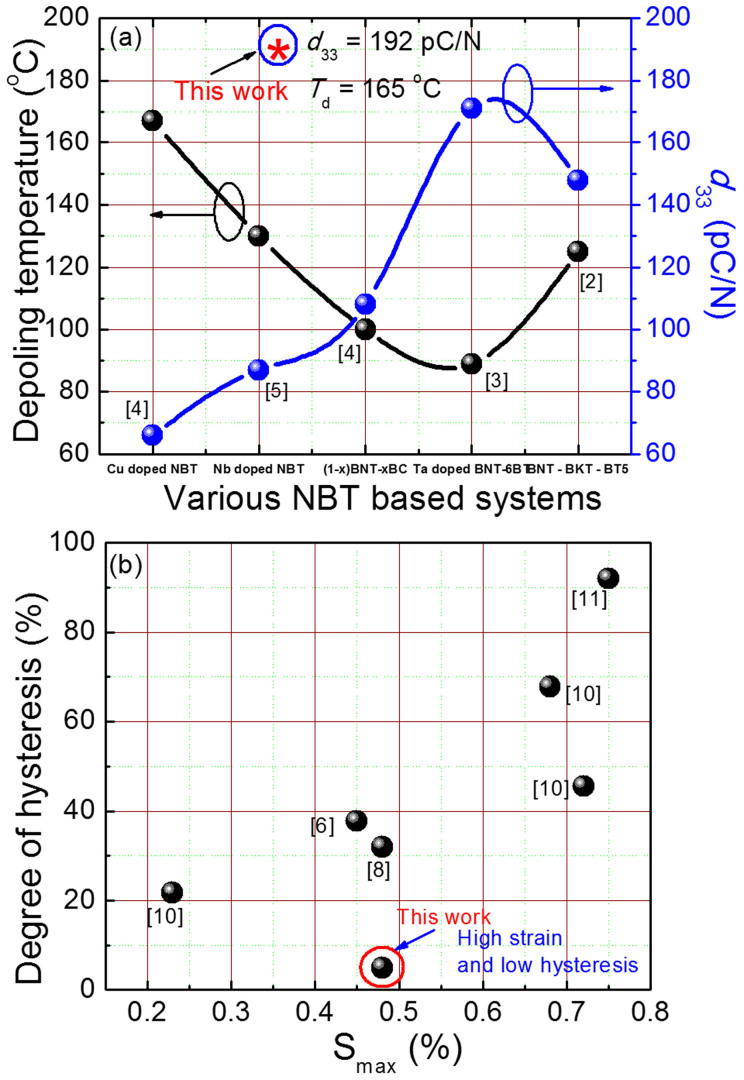
Enhanced piezoelectric response with high temperature stability and giant strain with ultra-low hysteresis. (a) Variation in depoling tempretaure and piezoelectric response of NBT based materials. The lines in Fig. 1(a) are just guide to the eye. (b) Degree of hysteresis ΔS/S_max_(%) versus maximum field induced strain (S_max_) for well-known material systems. Please note giant strain with small degree of hysteresis in textured KBT-BT-NBT system (This work).

**Figure 2:**
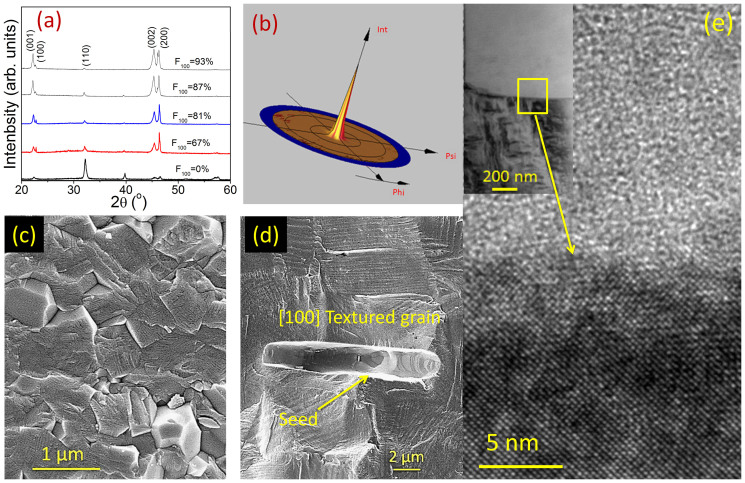
Texture analysis and microstructure. (a) XRD-spectra on samples having different degrees of texturing (as shown by Lotgering factor), (b) XRD pole figure of (002) planes of 93% textured specimen. (c) Cross-section SEM micrographs of (c) Randomly oriented KBT-BT-NBT (d) Textured KBT-BT-NBT specimen, (e) HR-TEM image of the interface of the BT seed and textured grain. The inset of Fig. 2 (e) shows low resolution image of the interface of textured grain and BTO interface.

**Figure 3:**
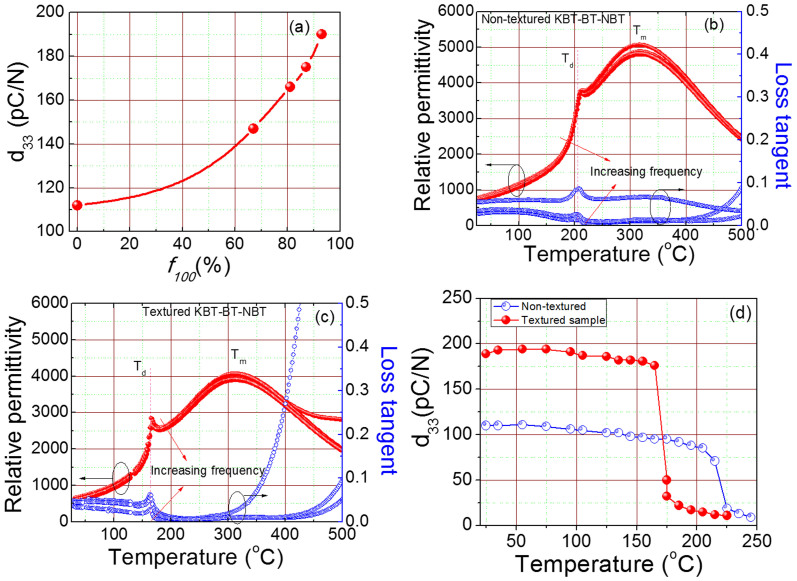
Piezoelectric and dielectric properties. (a) Longitudinal piezoelectric constant (*d*_33_) as a function of degree of texturing. (b) Relative permittivity and loss tangent versus temperature at various frequencies (1, 10, and 100 kHz) for: (b) Non-textured, (c) textured KBT-BT-NBT, (d) Temperature dependence of *d*_33_ for textured and non-textured samples. Please note: solid lines are just provided here as a guide for the eye.

**Figure 4:**
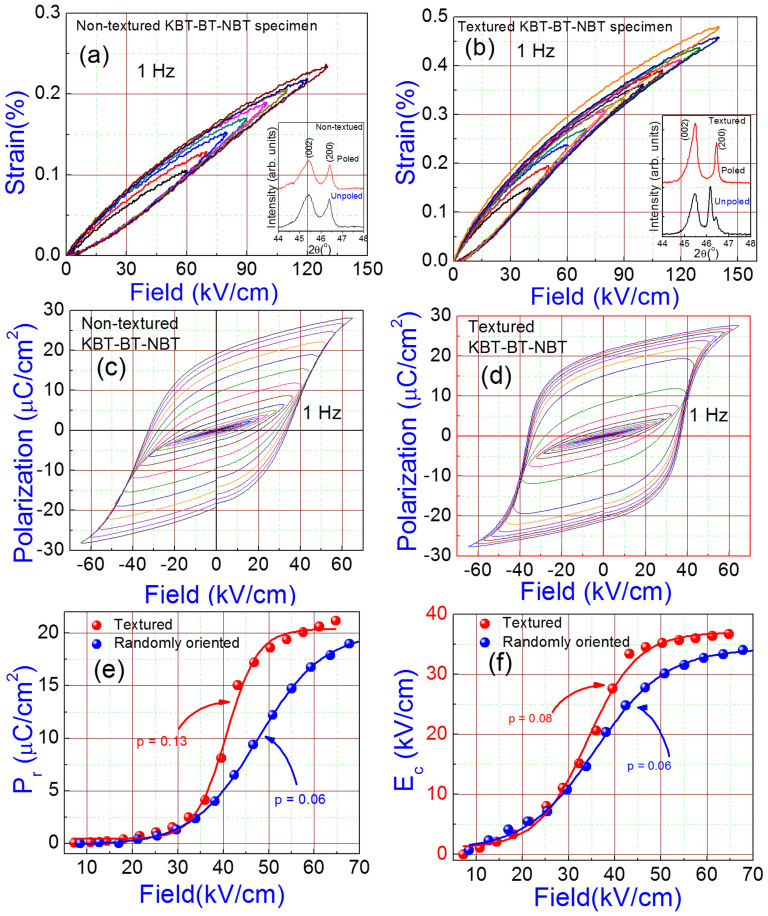
Giant *E*-field induced strain with ultra-low hysteresis and high remnant polarization. Unipolar strain versus *E*-field (*S*-*E*) plots for: (a) non-textured, (b) textured system. Insets of 4(a) and (b) show magnified view of (200) Bragg reflections of the XRD-spectra recorded on textured and non-textured samples, respectively. *P-E* hysteresis loops for: (c) non-textured, (d) textured system. (e) Remnant polarization (*P*_r_) versus *E*-field for non-textured and textured. (f) Coercive field (*E*_c_) versus applied *E*-field for non-textured and textured material. The solid lines in (e) and (f) are the fitted curves to the experimental data.

In order to measure the *E*-field induced strain in non-textured and textured KBT-BT-NBT systems, we performed strain versus *E*-field (*S-E*) measurements at 1 Hz at room temperature (Fig. 4a-b). The non-textured and textured specimens showed maximum values of *E*-field induced strain (*S*_max_) ~ 0.23% and 0.48%, respectively. Interestingly, the textured KBT-BT-NBT material exhibited a slim *S-E* loop with only 5% degree of hysteresis (Fig. 1(b)), which is an important property for actuator applications. This giant *E*-field induced strain (~0.48%) with ultra-low degree of hysteresis and depoling temperature as high as 165°C are some of the best values demonstrated for any lead-free piezoelectric. In order to estimate the energy dissipated during hysteresis measurements in the textured and non-textured ceramics, we calculated the area of the loop using the corresponding unipolar *P-E* hysteresis loops (Fig. S4 in supplementary information) at *E*_max_ ~ 130 kV/cm. We obtained 0.12 J/cm^3^ and 0.14 J/cm^3^ energy dissipated during unipolar hysteresis measurements for textured and non-textured samples, respectively. It is interesting to note that the textured samples with the dissipated energy of 0.14 J/cm^3^ at 130 kV/cm exhibited 0.44% strain as compared to 0.23% in non-textured specimen. This demonstrates the enhanced *E*-field induced strain with comparatively lower energy dissipation for textured sample. The high value of the *E*-field induced strain along with a small degree of hysteresis and high temperature stability (>160°C) clearly indicates the high practical relavance of the grain-oriented KBT-BT-NBT ceramics.

Large *E*-field induced strains have been associated with increase in the switching of non-180° domains^11^. Furthermore, we recorded the room temperature XRD-spectra of poled and unpoled textured and non-textured samples at 25°C in order to investigate the non-180° domain switching. The insets of Figs. 4a-b show an enlarged view of the {200}_c_ Bragg reflections. From these plots, one can notice only a slight difference in the shape and relative intensity of the (200)_c_ and (002)_c_ reflections for poled and un-poled conditions in non-textured sample. However, in the case of textured specimens, we observed a much more notable variation in the shape and intensity of the (200)_c_ and (002)_c_ reflections under *E*. These prominent changes in the intensity of the reflections due to poling of textured samples suggests a significant switching of non-180 domains^23^. We expect such phenomenon to make significant contributions towards enhanced *E*-field induced strain in textured samples. The volume fraction of non-180° domain (*V*_002_) can be estimated as: 
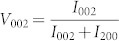
, where I_200_ and I_002_ are the fractions of the integrated intensities of the (200)_c_ and (002)_c_ peaks, respectively^24^. We observed 20% change in *V*_002_ due to poling in textured sample as compared to only 0.4% for non-textured ones. The domain switching in a polycrystalline ceramic material is complex as the switching in one grain could be constrained by neighboring grain having different crystallographic orientation^14^,^18^. Therefore, the polycrystalline tetragonal sample exhibited limited non-180° domain switching, and consequently, smaller unipolar strain due to intergranular mechanical constraints^18^. On the other hand, [001]_c_ crystallographic orientation in textured tetragonal specimen facilitates non-180° domain switching^18^. The high degree of non-180° domain switching, observed in the present work, is in agreement with the theoretical predictions^18^.

Furthermore, we performed polarization hysteresis (*P*-*E*) measurements on the textured and randomly axed KBT-BT-NBT samples at 1 Hz (Fig 4c-d). A high coercive field resulted in square-type *P*-*E* loops for both types of samples. The remnant polarization (*P*_r_) of textured ceramics was higher than that of randomly axed ones. Figs. 4e-f show comparative *P*_r_ and the coercive field (*E*_c_) as a function of *E* for non-textured and textured specimen, respectively. The solid lines in these figures are the fitted curves (Sigmoidal Dose-response curve with variable hill slope ‘*p*') to obtain the center and slope of the curves. The slopes (*p*) obtained by fitting *P*_r_ (*E*) plots for textured and non-textured specimen were 0.13 and 0.06, respectively. Also, we observed that the center of the curve (obtained by this fitting) was shifted towards lower fields (40.6 kV/cm) in textured specimen as compared to non-textured specimen (47.6 kV/cm). The higher value of the slope in textured specimens suggests an occurence of coherent domain switching at comparatively lower *E*-fields than that of non-textured ones. Also, lowering of the center of a curve for textured specimens suggests that domain alignment starts occuring at relatively lower *E*-field.

Using theoretical calculations, researchers demonstrated phenomenon of the domain wall broadening under *E*-field^25^,^26^,^27^. (for more information please refer to the supplementary document) The domain wall broadening effect was considered similar to the effect of the small domain size as smaller domain size results in higher density of domain walls^28^. The domain wall broadening and resulting high density of domain walls has been proven to result in enhanced piezoelectric response^18^. We believe this to be the operating mechanism for the enhanced piezoelectric response of the textured ceramics studied here.

### Domain structure analysis

**Figure 5:**
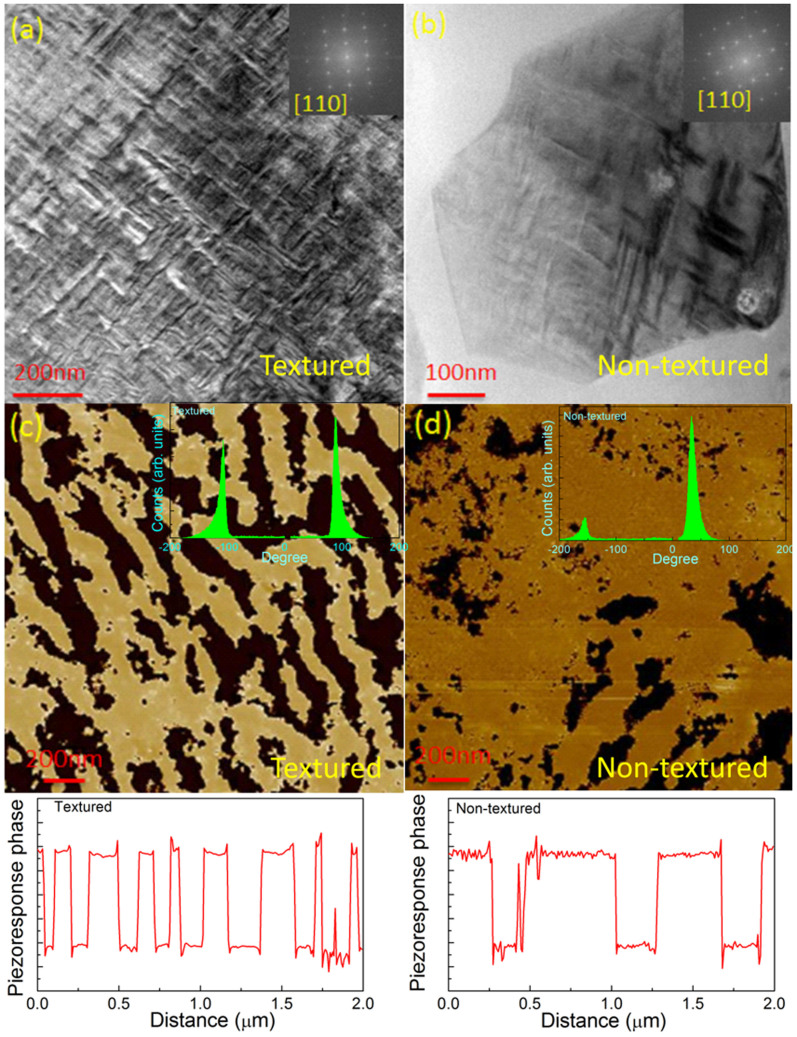
Small domain size in textured samples as compared to non-textured counterpart. Bright field TEM image of domain structure from [110]_c_ zone axis for (a) textured and (b) non-textured sample. Piezoresponse force microscopy image for (c) textured and (d) non-textured specimens with insets showing phase histograms. The bottom of the PFM image shows corresponding piezoresponse phase profile generated from the line scan across the domains.

In order to understand the domain structure, TEM and PFM measurements were peformed. Figs. 5a-b show bright field TEM images for the textured and non-textured specimens, respectively. The electron beam was parallel to the [110]_c_ orientation. In the case of textured specimens a wide spread of periodic tweed like domain morphology having a size of about 10–50 nm was observed. The tweed type domain morphology contribute significantly towards the enhanced field induced strain^29^. However, non-textured samples were found to show larger lamellar type domains. The domain formation occurs to minimize the elastic energy^30^. However, in case of textured sample, the total elastic enenrgy is expected to be influenced by the contributions from the seed templates and difference in grain sizes. These perturbations in elastic energies are such that they results in finer domain size in textured specimen. A narrower distribution of finer sized domains can be an important factor for enhanced piezoelectric response in textured samples as discussed above. Fig. 5c-d show the resonance-enhanced piezoresponse force microscopy phase images demonstrating domain structure of textured and non-textured KBT-BT-NBT specimens, respectively. The insets in Fig. 5c-d are the histograms of the corresponding PFM phase images. The peaks in the histograms indicate the distribution of domains with different orientation. One can clearly observe the nearly equal and narrow distribution of intensities in textured specimen as compared to non-textured counterpart. The textured specimen had a characteristic domain width smaller than that of the non-textured ones, consistent with the TEM results. This is quite visible in the line scan plot of the piezoresponse phase across the PFM images of textured and non-textured specimens. Moreover, the textured specimens exhibted more ordered domains, relative to non-textured ones; this facilitates coherent domain switching in textured materials. The PFM phase images provided in Fig. 5c-d depict representative domain structure for both the samples. Larger scans of ferroelectric domains for textured and non-texrured scans are shown in supplementary information (Fig. S7). The piezoelectric anisotropy along with higher mobility of domian wall contributes towards the enhanced piezoelectric response of the textured ceramics.

## Conclusions

We successfully synthesized highly textured lead-free KBT-BT-NBT piezoelectric materials. Textured specimens exhibited a ~70% increase in the low field longitudinal piezoelectric response, and more than 200% increase in the *E*-field induced strain with a ultra-low degree of hysteresis. The textured samples exhibited higher remnant polarization than that of their non-textured counterparts. The textured specimens had depoling temperatures of more than 165°C revealing a higher stability temperature for lead-free piezoelectric systems. The grain-oriented K_0.5_Bi_0.5_TiO_3_-BaTiO_3_-Na_0.5_Bi_0.5_TiO_3_ (KBT-BT-NBT) system is promising for high temperature actuator and sensor applications. This work opens new possibility to achieve high performance lead-free piezoelectric materials.

## Methods

### Sample preperation

Synthesis of (1-x)(K_0.5_Bi_0.5_TiO_3_-BaTiO_3_)-xNa_0.5_Bi_0.5_TiO_3_ with KBT: BT = 2:1 (denoted as KBT-BT-NBT) lead-free piezoelectric ceramics was performed using conventional solid state reaction method. The value of x was taken as 0.7, 0.8, 0.9 and 1.0. In supplementary information, Fig. S1 shows the optimized properties for these compositions. For texturing, BaTiO_3_ (BT) platelet seeds (Fig. S2) were synthesized using topochemical microcrystal conversion method^15^,^31^. The detailed process for the synthesis of grain oriented ceramics is provided elsewhere^15^. The surface morphology of the sintered samples was observed using a LEO Zeiss 1550 (Zeiss, Munich, Germany) scanning electron microscope (Figure S3).

### XRD measurements

We used Philips Xpert Pro x-ray diffractometer (Almelo, The Netherlands) for recording XRD-spectra at room temperature. The degree of orientation was determined from the XRD pattern in the range of 2θ = 20–60° by Lotgering's method^32^. The Lotgering factor *f* is defined as the fraction of area textured with required crystallographic plane using the formula Lotgering Factor 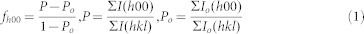
where I and I_o_ are intensity of the diffraction lines (hkl) of textured and randomly oriented specimens, respectively. The XRD-pole figure was obtained using a Philips MPD high-resolution X-ray diffraction system, equipped with a two bounce hybrid monochromator and an open three-circle Eulerian cradle.

### Electrical and piezoelectric characterization

For electrical measurements, the samples were cut in to rectangular shapes (3 mm × 2 mm) having thickness of 0.7 mm. For the electrical measurement, we applied silver electrodes on the flat faces of the rectangular specimen and then fired at 650°C for 30 minutes. Subsequently, we poled samples at 5 kV/mm at 60°C in the silicone oil bath and measured longitudinal piezoelectric constant (*d*_33_) using *d*_33_ meter (APC International, Ltd) based on Berlincourt method. We further measured dielectric constant and tangent loss factor as a function of temperature at selected frequencies using HP 4284A LCR meter connected to a computer-controlled high temperature furnace. In order to measure unipolar strain versus *E*-field plots at a frequency of 1 Hz, we used a modified Sawyer-Tower circuit and a linear variable differential transducer driven by a lock-in amplifier (Stanford Research, SR850). The degree of hysteresis Δ*S*/*S*_max_ (%) was calculated from the *S-E* loops. Δ*S* is the the deviation in strain during the rise and fall of the field. The energy dissipated in *S-E* hysteresis was obtained from the area of the corresponding unipolar *P-E* loop measured at 1 Hz as shown in Fig. S4. Polarization–electric field (*P-E*) hysteresis measurements were conducted by using the modified Sawyer-Tower bridge Precision II (Radiant Technologies).

### Transmision electron and Piezoresponse force microscopy

In order to prepare the electron transparent TEM specimens, we used standard grinding and ion-milling method. For conducting transmission electron microscopy, we used a FEI Titan 300 microscope. The TEM images of the domain morphology at various magnifications for textured and non-textured samples are shown in Fig S5 and S6, respectively. We used a scanning probe microscope (Bruker Dimension Icon, USA) coupled with conductive platinium coated silicon cantilvers to perform the Piezoresponse force microscopy (PFM) on the textured and non-textured samples. Large area PFM scans are shown in Fig. S7.

## Author Contributions

D.M. and S.P. conceived the idea and wrote the paper. D.M. synthesized textured sample and performed piezoelectric/ferroelectric measurements along with the TEM characterization. Y.Z. performed PFM measurements. Y.Y. helped in formatting the manuscript and recording SEM results. Y.W. performed *S*-*E* and XRD-pole figure measurements. J.L. and D.V. supervised the *S*-*E* and XRD-pole figure measurements along with providing suggestions on the text of the manuscript. S.P. supervised the research. All authors discussed the results and commented on the manuscript.

## Supplementary Material

Supplementary Information Supplementary Information

## Figures and Tables

**Table 1:** List of strain values and transition temperatures for well-known lead-free piezoelectric materials

Sample	Strain (%)	*T*_c_ (°C)	*T*_d_(°C)/*T*_ot_ (°C)
KBT-BT-NBT (This work)	0.48	300	165
(Na_y_,Bi_z_)Ti_1−x_O_3__(1−x)_-xBaTiO_3_^6^	0.48	300	100
(K_0.5_Na_0.5_)_0.98_Li_0.02_NbO_3_(Textured)^7^	0.1	425	143
BNT-BT-KNN2^8^	0.45	250	100
Na_0.5_Bi_0.5_TiO_3_–6BaTiO_3_ single crystals^9^,^10^			
(air annealed)	0.23	280	99
(Oxygen annealed)	0.72	280	99
(Vacuum annealed)	0.52	280	99
BaTiO_3_ [100] aged single crystal (Based on symmetry-conforming point defects)^11^	0.75	120	---

***T*_ot_**: Orthorhombic to tetragonal transition.
