# Supplementary material for: Giant strain with ultra-low hysteresis and high temperature stability in grain oriented lead-free K0.5Bi0.5TiO3-BaTiO3-Na0.5Bi0.5TiO3 piezoelectric materials
Source: Sci Rep. 2015 Feb 26;5:8595. doi: 10.1038/srep08595 (PMC4341219; doi:10.1038/srep08595)
Supplement: Supplementary Information [file srep08595-s1.pdf]

Giant strain with ultra-low hysteresis and high temperature stability in grain oriented *lead-free* $K_{0.5}Bi_{0.5}TiO_3$-$BaTiO_3$-$Na_{0.5}Bi_{0.5}TiO_3$ piezoelectric materials: supplementary information

Deepam Maurya[1], Yuan Zhou[1], Yaojin Wang[2], Yongke Yan[1], Jiefang Li[2], Dwight Viehland[2], and Shashank Priya[1*]*

[1]Bio-inspired Materials and Devices Laboratory (BMDL), Center for Energy Harvesting Materials and Systems (CEHMS)
Virginia Tech, 24061 USA
[2]Department of Materials Science and Engineering, Virginia Tech, Blacksburg, VA 24061,USA

Keywords: lead-free, ferroelectric domains, grain-oriented piezoelectric materials.

*Corresponding author E-mail: mauryad@vt.edu, spriya@vt.edu

Figure-S1 shows the piezoelectric and dielectric response of various compositions prepared across the MPB of $K_{0.5}Bi_{0.5}TiO_3$-$BaTiO_3$-$Na_{0.5}Bi_{0.5}TiO_3$ system. From this plot, one can clearly notice the enhancement in the piezoelectric response of the MPB composition. However at MPB, the enhancement in the electromechanical properties has been found to accompany lowering in depoling temperature (T_d). Therefore, for texturing process, we chose composition away from the morphotropic phase boundary (MPB) is x=0.8 in (1-x)(KBT-BT)-xNBT system with KBT:BT=2:1. This composition will be referred as (KBT-BT-NBT).

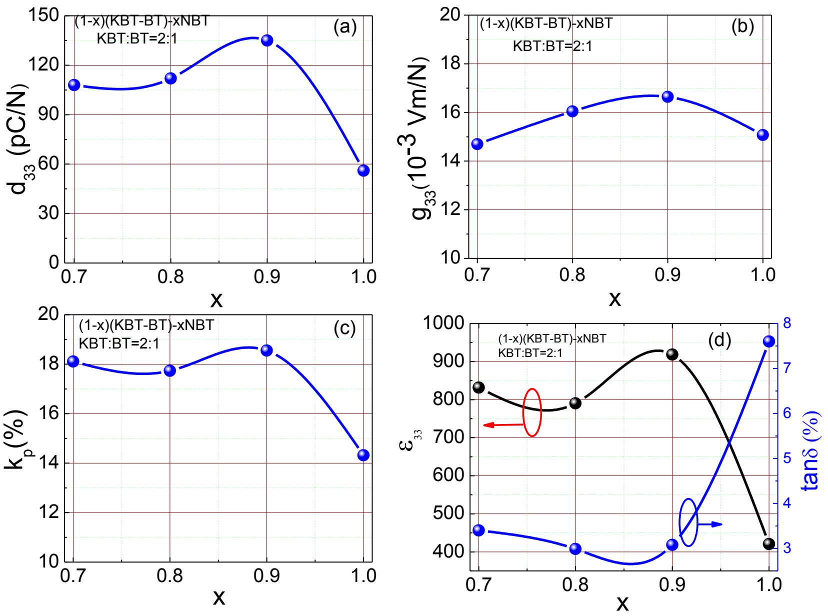

Figure S1 | Piezoelectric and dielectric response of various compositions of lead-free piezoelectric ceramics (1-x)($K_{0.5}Bi_{0.5}TiO_3$-$BaTiO_3$)-x$Na_{0.5}Bi_{0.5}TiO_3$ system with KBT: BT = 2:1 having x ~ 0.7, 0.8, 0.9 and 1.0. Compositional dependence of **(a)** piezoelectric constant (d_{33}), **(b)** voltage constant (g_{33}), **(c)** planar electromechanical coupling (k_p), **(d)** dielectric constant (ε33) and loss tangent factor (tanδ).

In order to synthesize textured samples, we need shape anisotropic seed templates having similar crystallographic parameters. For this, we synthesized anisotropic shaped BaTiO3 seed templates usign micrystal conversion method. Figure- S2 shows the SEM micrographs of the $BaTiO_3$ templates thus synthesized. We aligned these template seeds in the powder matrix of the materials to be textured and process at 1150°C upto 50h to get textured KBT-BT-NBT piezoelectric ceramics.

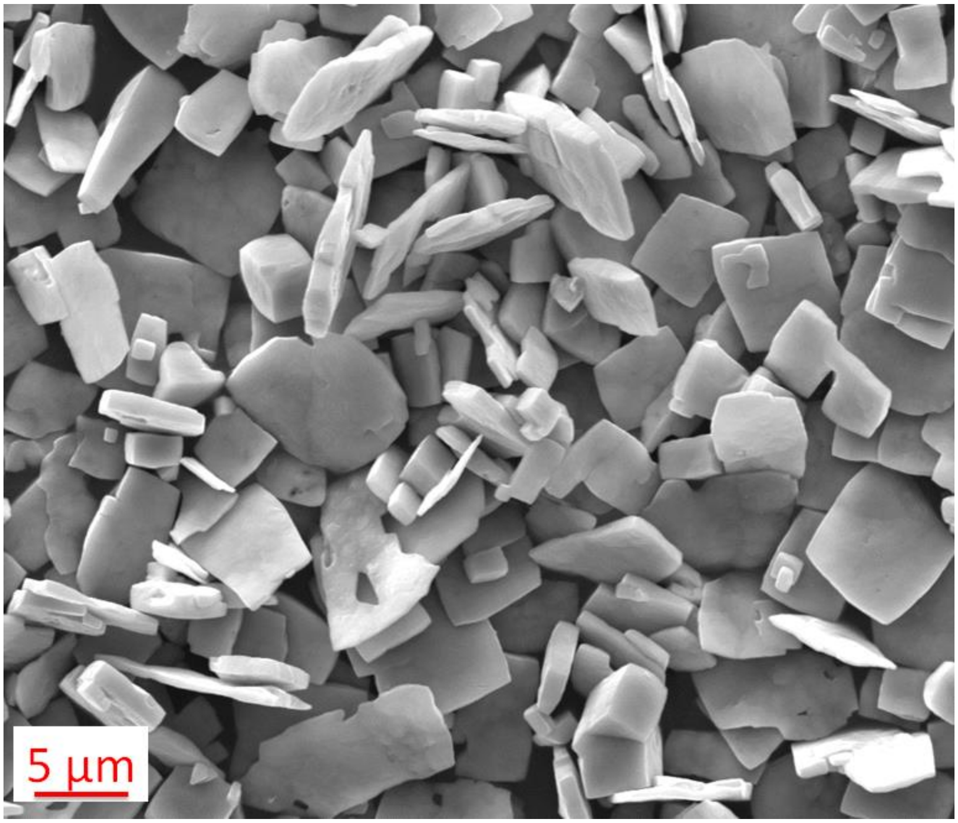

Figure S2 | Seed platelets. SEM micrograph of the $BaTiO_3$ platelets used as template seeds for texturing.

Figure-S3 shows the SEM micrographs of non-textured and textured sample. From this figure we can clearly notice the difference in the grain size of non-textured and textured specimens as the average grain size of non-textured sample was much smaller.

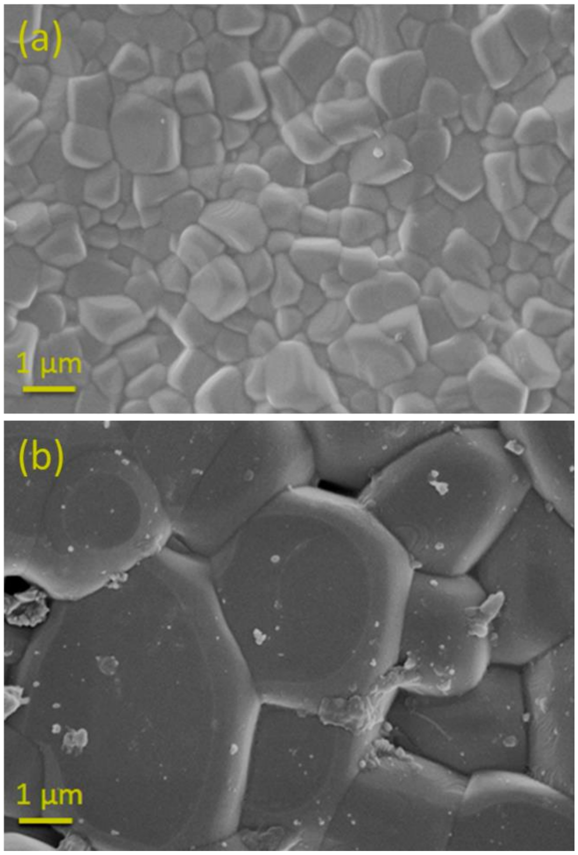

Figure S3 | Top surface microstructure. SEM micrographs of **(a)** non-textured and **(b)** textured sample.

The energy dissipated in S-E hysteresis was obtained from the area of the corresponding unipolar *P-E* loop measured at 1 kHz as shown in Fig. S4. The area of the loop was calculated by fitting 5th order polynomial to the upper and lower branch and integrating the difference of the area under the curves. Figures S5*a-d* show the bright field TEM image of the domain structure for textured sample at various magnifications from $[110]_c$ zone axis. Figures-S5*c-d* show the HR-TEM images near domain walls. The insets of Figures S5*c* and *d* indicate corresponding FFT patterns. The magnified image of a spot with streaking (In the inset) from the FFT pattern indicates the distribution in the intensity due to the presence of domain walls.

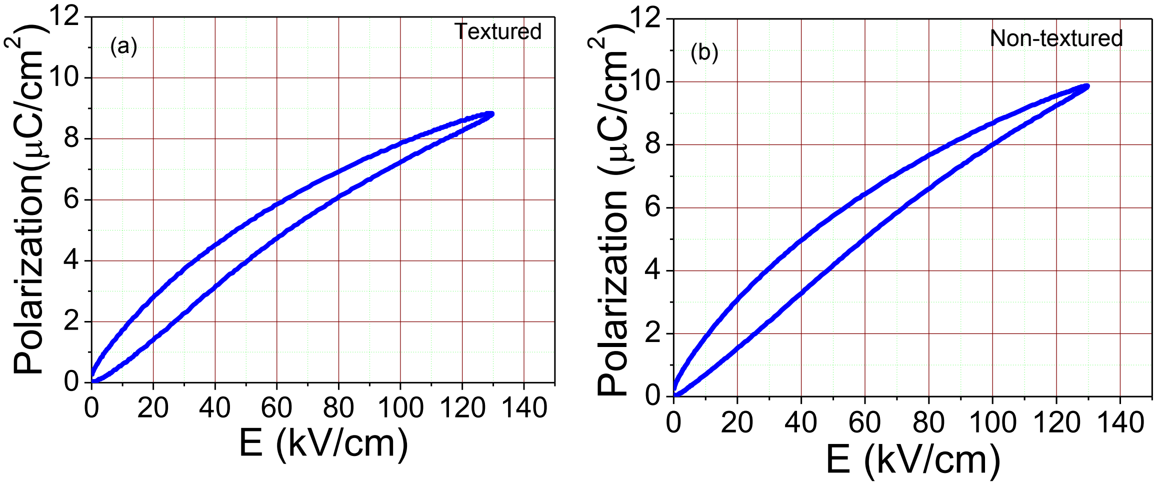

Figure S4 | Unipolar polarization hysteresis. Room temperature unipolar polarization hysteresis plot at 1kHz for **(a)** textured, **(b)** non-textured sample.

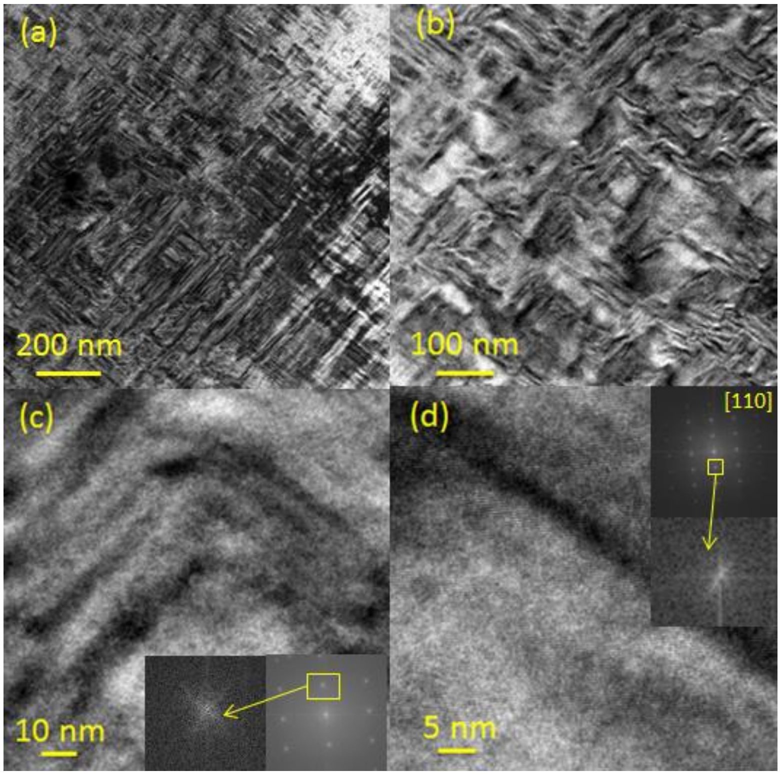

Figure-S5 Domain morphology in the textured specimen. (a)-(b) Bright field TEM image of domains in the textured KBT-BT-NBT. **(c)- (d)** HR-TEM image of domains in textured sample.

Figure S6*a* show the bright field TEM image of the domain morphology from [110] orientation. Figure S6*b* show the HR-TEM image of the area near the domain wall. The presence of streaking in the magnified view of the spot from FFT pattern is due to the presence of the domain wall.

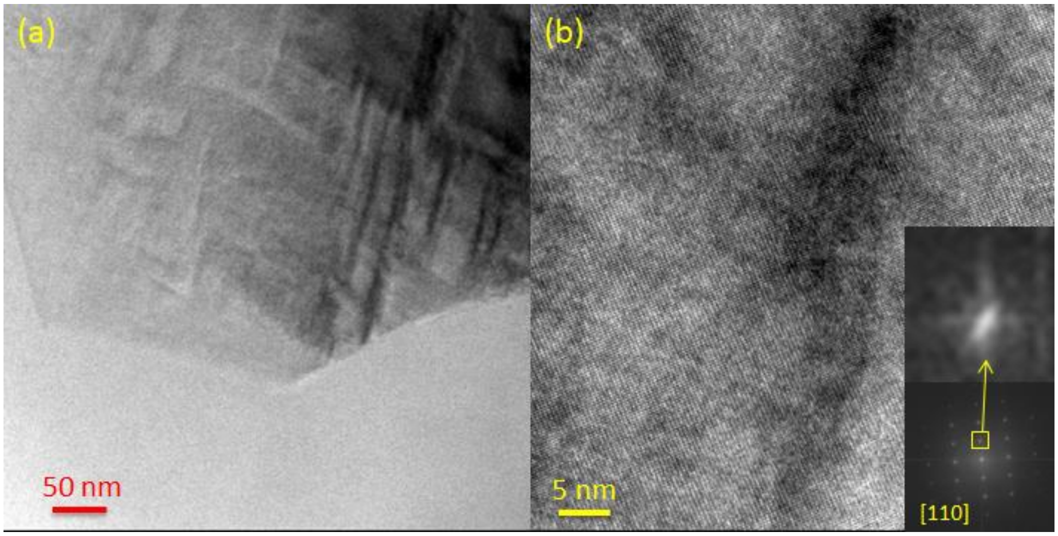

Figure-S6 Domain morphology in the non-textured specimen. (a) Bright field TEM image of domain morphology in the non-textured KBT-BT-NBT system. (b) HR-TEM image of the region near domain wall corresponding to Fig. S6 (a).

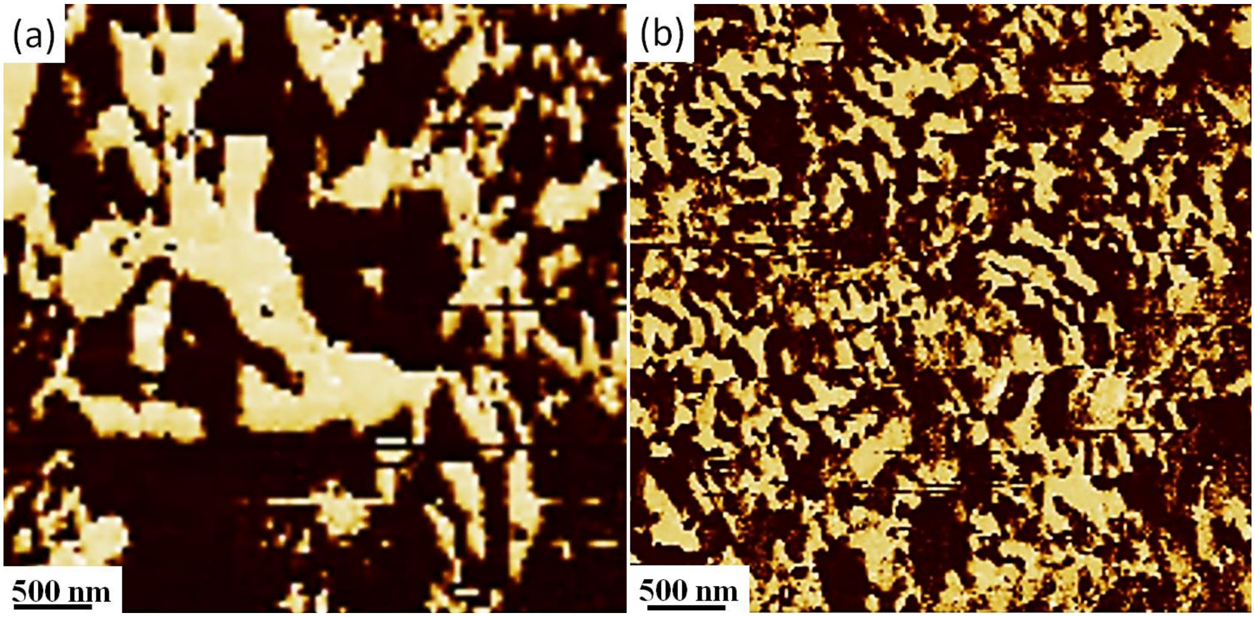

Figure-S7 Long range domain morphology probed through Piezoresponse force microscopy (PFM). PFM image of domains for (a) textured and (b) non-textured specimens.

The PFM images presented in Fig. S7 show domain morphology over larger scanned area. One can clearly observe the difference in the domain morphology of textured and non-textured samples. The smaller domain size plays a major role towards enhanced piezoelectric response of a system.

Effect of smaller domain size on piezoelectric response

The total free energy, of a multi domain ferroelectric single crystal (having polarization field **P**(**r**)), under electric field $\mathbf{E}^{ex}$ can be given as[1,2,3]:

$$F = \int d^3 r \left[f(\boldsymbol{P}) + \frac{1}{2}\beta_{ijkl}\nabla_i P_j \nabla_k P_l - P_k E_k^{ex} \right] + \frac{1}{2}\int \frac{d^3 k}{(2\pi)^3} \left[\frac{n_i n_j}{\varepsilon_0} \widetilde{P_i}\widetilde{P_j^*} + K_{ijkl}\widetilde{\varepsilon_{ij}^0}\widetilde{\varepsilon_{kl}^{0*}} \right] \quad (1)$$

where, $f(\boldsymbol{P})$ is the local bulk free energy density represents the thermodynamic properties of stress-free homogeneous ferroelectric state,[1] β_{ijkl} is the energy contribution from the polarization gradient in domain wall, ∇_i is the gradient operator, and $K_{ijkl} = C_{ijkl} - n_m C_{ijmn} \Omega_{np} C_{klpq} n_q$, $\Omega_{ik} = (C_{ijkl} n_j n_l)^{-1}$, C_{ijkl} represents the elastic modulus.[1] The Fourier transform of polarization distribution **P**(**r**) and strain distribution $\boldsymbol{\varepsilon}^0(\mathbf{r})$ is represented by **P**(**k**) and $\boldsymbol{\varepsilon}^0(\mathbf{k})$ respectively, and '*' represents the complex conjugate.[1] The time dependent Ginzbrug-Landau equation can be used to represent the electric field-induced phase transition and domain evolution[1, 2, 3]:

$$\frac{\partial P_i(\boldsymbol{r},t)}{\partial t} = -L_{ij} \frac{\delta F}{\delta P_j(r,t)} + \xi(\boldsymbol{r}, t) \quad (2)$$

where L_{ij} is the kinetic coefficient, $\xi(\boldsymbol{r}, t)$ is Gaussian-distributed Langevin noise term. Using equation (1) and (2), researchers observed domain wall broadening under applied E-field.[1] The domain wall broadening has been considered analogous to the reduction in domain size, which, in turn, results in enhanced piezoelectric response[4].

References:

1 Rao, W.-F. & Wang, Y. U. Domain wall broadening mechanism for domain size effect of enhanced piezoelectricity in crystallographically engineered ferroelectric single crystals. *Applied Physics Letters* **90**, 041915 (2007).

2 Semenovskaya, S. & Khachaturyan, A. G. Development of ferroelectric mixed states in a random field of static defects. *Journal of Applied Physics* **83**, 5125-5136 (1998).

3 Li, Y. L., Hu, S. Y., Liu, Z. K. & Chen, L. Q. Phase-field model of domain structures in ferroelectric thin films. *Applied Physics Letters* **78**, 3878-3880 (2001).

4 Wada, S., Yako, K., Kakemoto, H., Tsurumi, T. & Kiguchi, T. Enhanced piezoelectric properties of barium titanate single crystals with different engineered-domain sizes. *Journal of Applied Physics* **98**, 014109 (2005).
